# Interactive freehand sketching as the means for online communication of design intent in conceptual design conducted by Brainwriting

**DOI:** 10.1007/s12008-020-00745-x

**Published:** 2020-11-07

**Authors:** Sergio Rizzuti, Luigi De Napoli

**Affiliations:** Department of Mechanical, Energy and Management Engineering (DIMEG), University of Calabria, Ponte Pietro Bucci 46/C, 87036 Rende, CS Italy

**Keywords:** Sketching, Product design, Brainwriting, Interactive design, Communicate design intent, Semiology

## Abstract

Sketching is becoming an irrelevant activity of engineering studies. The availability of many software that aids designers in all phases of design, not only analytic but synthetic, push technicians, designers to use such tools, giving up the employment of a simple pencil and eraser on a sheet of paper. The productivity of software tools is obliged to speed and manage the whole design process; even freehand sketching remains the fundamental means to communicate the first ideas immediately. During Brainwriting sessions, the ability to explain by sketches first elaborations of a possible solution, that must be understood by co-designers, is the first step that allows more fruitful discussion and immediate adjustment towards a quick embodiment of valid proposals. The paper describes how such techniques has been introduced in the mechanical engineering curriculum. The case of study reports the experience of the Brainwriting online, which has been tested during lockdown due to the pandemic disease of COVID-19. Further in the paper it is suggested a new interpretation of the de Saussure general linguistic studies, in term of a communication that is associated to a drawing.

## Introduction

The need to exchange information around a problem is the prerequisite for trying to solve the problem. Designers typically employ sketches that can be done in different ways and using several tools: a simple pencil and an eraser on paper or tablet with sketch tools.

With the development of digital technologies, it has been tried to aid the freehand sketching of industrial products in the first phase of design, deploying sophisticated hardware and software tools [1].

First of all, there has been the try to replace the paper and pencil with the graphic tablet, a special stylus, or simply the mouse, remaining in the 2D sketch [2].

Subsequently, an attempt was made to switch from 2D to 3D design, also with the aim of directly obtaining translation into a 3D CAD models [3, 4].

In the first decade of the 2000s, an attempt was made to adopt freehand sketching tools in Virtual and Augmented Reality as a support to the designer in the conceptual design phase, with results that seemed promising [5–8].

However, many of these new tools have not found much success except in rare cases, in which the technological ability is combined with the manual one of the individual designer. Very often, however, in the industrial engineering design offices, the aim is to keep the classic techniques of freehand sketching, in order not to mortify and block the creativity of designers [9].

In any case, the characteristic aim of all these methods is to guarantee a quick and efficient way for transmitting information, employing standard graphical information, even 3D representation of hypothetic forms, or symbols to characterize them, or notes that specify their peculiarities.

A sketch does not require great precision. Obviously, good quality can explain better the initial intent, but this is not mandatory. Furthermore, a certain level of “imprecision” can allow designers of enlarging the context and the content of the proposal, a sort of hidden learning or misunderstanding that can suggest new ways to follow.

In recent years, a reconsideration of freehand drawing is taking place at various levels. Many studies have shown that, in all STEM disciplines from elementary school to the highest levels of education, hand sketching brings many benefits in learning [10–12]. Furthermore, despite the increasing development of computer-aided techniques, there is a revival of freehand drawing in engineering, particularly in mechanical engineering [13].

The return to this practice is mandatory because of its centrality in concept generation of a product, both as a new one or as a redesign, when creativity needs the right means to reveal itself [14].

To foster creativity in concept generation, several methods have been conceived and several variations have been proved with different emphasis on the work, made alone or in group. The methods that not employ the classic Obsborn’s brainstorming approach are: Brainsketching (Brainwriting), Gallery, C-Sketch, 6–3–5 methods [15].

The method employed in this paper is a mix of these and it has been adopted in concept generation in the courses of Product Design and Development [16].

## Need for enhancing interactive sketching ability

The sketching ability must be acquired along with the engineering studies. Engineering drawing courses, the typical courses of drawing, in which students are involved, are oriented to form professional competences, to communicate effectively and efficiently the nature of a device or each singular component.

They are based on the acquisition of many rules and the right interpretation of symbols. Along time the symbols that have been added to a simple drawing are becoming terribly great. The knowledge is becoming almost distort moving from the essential definition of a form to its characterization in terms of precision and assemblability. The author’s opinion is that more attention should be devoted to learn techniques and acquire competencies on sketching, that allows students to quickly elaborate new forms or variations, employing poor tools. Only after this first basic step, it is reasonably possible to understand and discuss around device functionalities that must be employed in detail design.

Sketching by drafts can be performed in two ways: 2D and 3D. The tasks are a little different because the intent is strictly related to the kind of information that must be communicated.

### Sketch 3D

During conceptualization, a sketch that gives a global vision of a device or a set of components allows readers to easily collocate the device in space and grasp the essential aspect of the idea.

The great difference that distinguishes mechanical engineers and architects is the dimension of the item thought. Architects are more familiar with perspective representation because the objects on which they are involved have dimensions that an observer sees by a wide field of view and this induces towards representation with lines converging towards vanishing points.

Mechanical Engineers typically apply axonometric representation, even oblique or orthogonal, and the draft is elaborated more quickly. The amount of information is identical to perspective in term of shape, but it allows technicians to measures dimensioning.

### Sketch 2D

When a component must be manufactured or the functional aspects must be verified in an assembly, a 2D sketch allows stakeholders to easily draw, add, erase or modify elements to pursue requirements. This phase is generally associated with something that has reached a certain degree of detail, or when a reverse engineering study is started. This kind of sketches requires more precision and, if performed manually, millimetre paper can aid designers.

## Interactive sketching during Brainwriting

Freehand sketching is the most powerful, almost the unique, technique to develop and show creative idea during concept phase of product design [15].

Over the year, Brainwriting has been proved by present authors as the best tool for expressing innovative ideas in team [16].

Brainwriting is a method by which every member of a team can freely explain his/her ideas and interact with and integrate those proposed by the others. To be performed, Brainwriting requires a group of people that must not be too big (ranging from three to a maximum of six people) sitting around a table.

The method is divided into two phases. In the first phase, each member is free to explain his/her ideas on a sheet of paper (typically an A3 format) with drafts, notes and/or symbols, without interaction with the others, and emphasizing three aspects of a concept. After a certain amount of time (typically 10–15 min), each member passes the sheet of paper to his/her colleague on the right and continues to add positive suggestions to the ideas previously expressed by the others. In the second phase, each team shows all material along a gallery and discusses the design alternatives proposed, and the discussion involves the team altogether.

Among the techniques that allow the exhibition of creative ideas, Brainwriting is the one that most allows each member of the team to express freely his/her own thought.

**Fig. 1:**
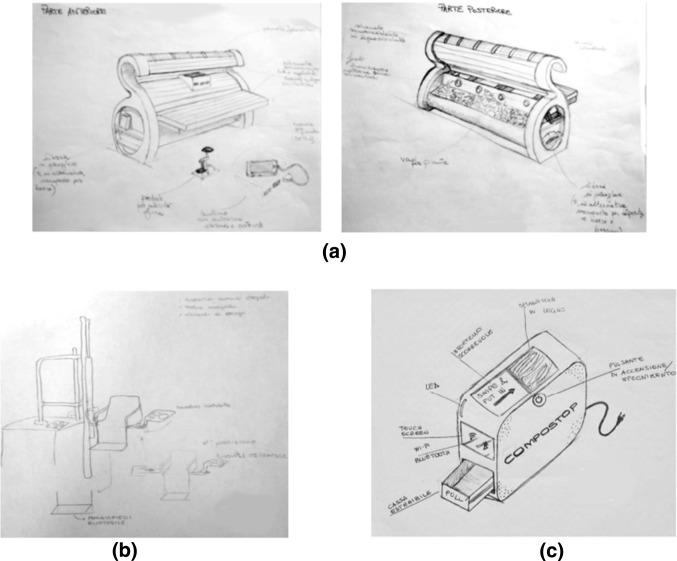
Sketches of concepts generated in Brainwriting sessions in past academic years: (**a**) a bench; (**b**) a chair for immersion of paraplegic in water to take a bath; **c** a domestic composter

Figure 1 shows some examples of the sketches made by team of students, during Brainwriting sessions in the course of Product Design and Development. The two sketches of Fig. 1a show the concept of a smart bench with energy harvesting systems, in this case the aim was to show more details of the object and this required two points of view; Fig. 1b shows a system to allow people with walking difficulties to access swimming pools; Fig. 1c shows the concept of a domestic composter.

### de Saussure interpretation of Brainwriting

Ferdinand de Saussure [17] is the scientist that elaborated, almost a century ago, the basics of the modern linguistics and invented semiology. Following his approach, it is possible to interpret the relation between two people that would like to exchange ideas, by a drawing. In this case the sign substitutes the sound of the words that two people exchange when speech themselves.

**Fig. 2:**
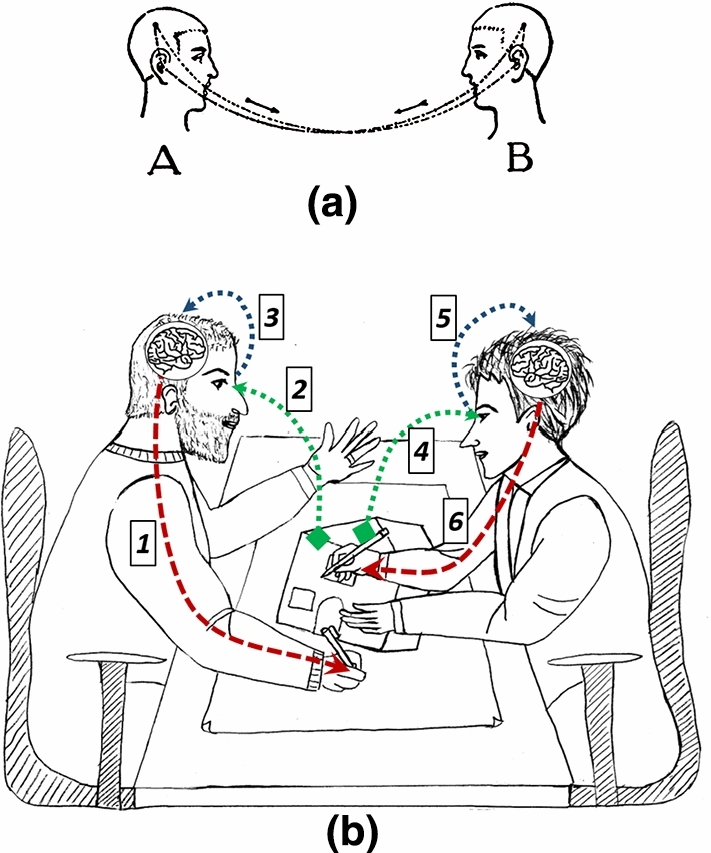
Exchange of information: (**a**) during a speech by de Saussure model; (**b**) by means of freehand sketching and Brainwriting method

Figure 2 shows two contexts in which an exchange of information happen.

In Fig. 2a there is the de Saussure representation of a conversation, in which two people, alternatively, speech. The first person (A) speeches and the sound reaches the ears of the second one (B), then the sound is elaborated by his/her brain [18]. Then this one (B) answers and the sound of his/her voice hit the ears of the first one (A) and the sounds are elaborated by his/her brain. The arrows show the interactivity during a speech. Figure 2b depicts this situation in which the exchange of information is done by a drawing. Imagine that the person on the left has drawn something on the paper (red dashed line) (1). He/she has seen by eyes (green dashed line) (2) and controlled by brain (blue dashed line) (3) what has been sketched on paper. If the sketch has some “signified” for him/her then he/she can release the drawing. Then the person on the right can start now to see the sketch (green dashed line) (4), elaborate some thoughts (blue dashed line) (5) and intervenes on the sketch (red dashed line) (6). The exchange must happen in alternation, without co-operation in the same time. So freehand drawing becomes the most effective tool for communicating concepts between designers’ brains. The first phase of Brainwriting obliges that along this exchange there were no words exchanged by designers. Only the drawing content is the means by which communication happens. This phase stresses the communication only by drawings.

Really, the communication by drawings and sketches happens “diachronically”, because a drawing can be sketched in a time in one place and read in another time in another place.

The strength of drawings and sketches is related to the quality of the signs that are employed and if the drafts are sufficiently well done to be recognizable.

**Fig. 3:**
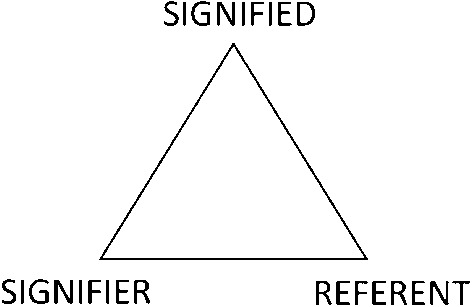
The semiotic triangle

In this, it is important to recall the de Saussure original research, which has been used as starting point for semiology. The semiotic triangle shown in Fig. 3 represents the relation that exists between a real object (REFERENT) and its meaning (SIGNIFIED) interpreted by signs (SIGNIFIER).

Understanding about a subject is possible only when the signs (SIGNIFIER) are recognized (SIGNIFIED) as related to something already known (REFERENT). But also during conceptual design what it is not already existent (a REFERENT) should be understood, rationally understanding its meaning (SIGNIFIED), reasoning on the signs (SIGNIFIERS) that are drafted on a device (paper or screen). And then the drawings can be considered associated to the solution of a problem to which designers are working on.

## Online Brainwriting

The experimentation described in this paper is based on the experiences done in the “Product Design and Development” course, included in the curriculum of the master degree in mechanical engineering at UNICAL (Italy). Being the course a typical case of Project Based Learning (PBL), because of COVID-19 emergency, all the activities have been redesigned in really short lapse of time, in the second semester of academic year 2019–2020. The shocking and somehow surprisingly astonishing novelty of this year has been the overturning of the way, by which lectures and group work have been carried out, because of the pandemic emergency.

For concept generation, a BrainWriting (BW) method, refined over the years [16], has been used. It is a variant of the 6–3–5 method, followed by a gallery method.

This year, classical Brainwriting has been turned on online, adapting, and redesigning the typical steps of the method by means of some online and offline tools.

Since many years, many tools have been developed to perform online brainstorming or other online teamwork activities. The peculiarity of Brainwriting is the freehand sketching activity, that is very difficult to virtualize.

In the present edition of the BW session, the following structure was adopted: the number of participants for each team was four, each team member had 15 min to design the initial concepts and 10 min for each successive improvement phase, where each participant marked improvements to the drawing made by other team members; the total duration of this session was fixed around 45 min; the timing has been checked by teachers and the participants had the denial of discussing each other. This year the first sketches were drawn on paper that was transferred as an image, by means of a JPEG file, to the other team components, following the surname alphabetic order. The improvements were added by Sketchbook®, opening the JPEG file on a different layer identified by a different colour, one associated to a different team member, in order that each component could have recognize his/her suggestions. Every time a step finished, each people saved the improvement in a TIFF file to save all suggestions. Each file was uploaded in a repository of Teams in such a way it could have been downloaded by another student, always following the order established. The TIFF format allows maintaining the structure of the levels, created in the phases of online Brainwriting. The BrainWriting session ended with the gallery of the concepts generated, within each group, to refine, classify, structure, and prepare all the materials produced, for the subsequent phase of the concept selection.

### Technologies for online Brainwriting deployment

The possibility to perform Brainwriting without the physical exchange of materials, as in case of an in-person laboratory, requires a set of tools. Mainly it is important to do the best choice of a collaboration tool that provides global, remote, and dispersed teams with the ability to work together and share information via a common space. Then it is necessary to identify a friendly tool for sketching or for annotation and able to maintain a data structure of all upgrades.

The choice was oriented towards Microsoft Teams® for the collaborative tool and Autodesk Sketchbook® for the second one. Probably other software solutions could be employed, but this is not important for the task of the paper.

It was created a “team” on Teams with the name of the course (Product Design and Development), then a series of hidden channels, one for each group of students or team, with a name identifying it. Every channel collected only the students of the team and the instructor. The instructor was present in all channels in order to follow the correct development of the session, jumping in each channel and talking with each group for any problem. In the channel “general”, the instructor has the possibility to interact with the whole class, at the beginning, to present the Brainwriting method or answer to questions that could emerge during laboratory.

The atmosphere was really intriguing and relaxing because each team worked in a very private space, while each member lived at his/her home. This has been the main aspect of homeworking.

The relation instructor-students was also reach, considering that many materials have been shown, while in-person the direct emotional contact can be considered the main characteristic.

At the end of this happening instructor asked students to write individually a brief essay in which tell the Brainwriting experience, which they lived in about 2 h. The reports had some similarities. Students, at the end of this experience, were really enthusiast of the method, because never supposed they would have been able to complete the task in a so short lapse of time. It was the first time they knew and employed Brainwriting. They all regret about some delays in the uploading and downloading the materials to and from Teams, due to net problem. They all explain that a better friendliness with Sketchbook would have allowed them to add more enrichments to the ideas, which they were asked to improve.

## Case study

In the academic year 2019–2020, the experiment has been performed in the fourth and fifth weeks of the semester schedule, which started in March 2020, just at the beginning of lockdown in Italy.

In the first three weeks, a set of activities were performed, led by the teacher. In the first week, the needs hidden into the product to be designed and related to the problem to be solved are exposed and examined. The students have been engaged on the theme “allow frequenting a gym for fitness in time of COVID-19”, which could materialize in a “mask”. An external online survey to understand better the real needs around this problem was autonomously organized by students. In the following week, the problem was analysed by functional analysis.

After this preliminary sessions, students had a week to enlarge the context of their knowledge around the problem with external research. This time was also necessary to incubate their own ideas. After that, a set of channels were organized in the Microsoft Teams® ambient in which each team, in their own channel, carried out the concept generation session.

**Fig. 4:**
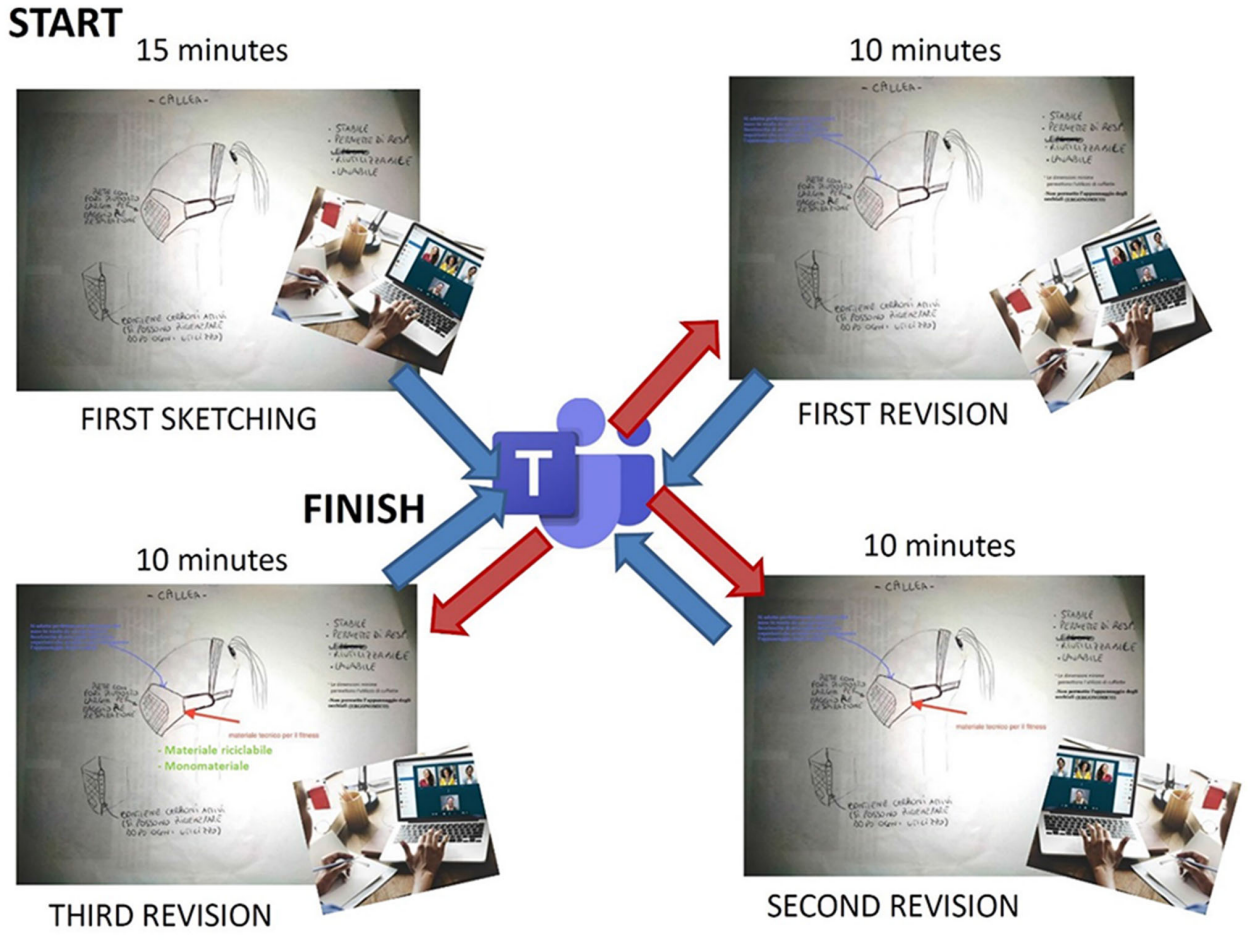
Evolution of one concept in the online Brainwriting session

In Fig. 4, the evolution of one concept in a group is shown. The four people, everyone at their home, worked on their own notebook. Everyone started their own freehand sketching. The figure depicts the sequence of exchanges that followed the work and the updates done on only one initial idea. But it must be taken into account that simultaneously four ideas was conceived and elaborated in the group. The blue arrows depicts the upload of the work on the repository, and the red arrows depicts the download from the repository.

At the end of the sequence the repository contained the files of all intermediate steps and the final concepts with the first sketch and all the notes and suggestions drawn with different colours, made by the other team components.

**Fig. 5:**
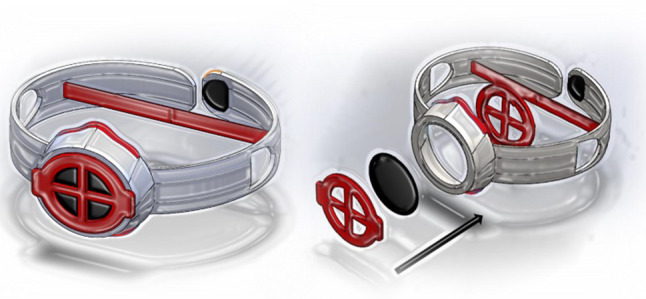
The cad model of a winning concept

In Fig. 5 is shown the embodiment of the concept, after the phase of the concept selection in a team.

## Conclusion

During this didactical experience the application of the Brainwriting online has been experimented. The need to pursue such kind of method not in person was due to the pandemic of COVID-19. Nevertheless the problematic effects of such disease, we have had the possibility to test such kind of communication. The tools that have been employed allowed to share efficiently the drawings among the students involved in conceptual design.

The degree of interaction between students did not degraded and a set of original ideas emerged during Brainwriting online.

Considering the first time of using Microsoft Teams and Sketchbook conjointly with the understanding of Brainwriting made slower the schedule previously defined, due to the downloading and uploading the JPEG files from and to the repository on Microsoft Teams.

Despite the students’ initial skepticism towards an online group activity like the one just described, the result was greatly appreciated by many of them, even some declared themselves enthusiastic and amazed.

Quoting a student: “At the beginning, I thought it was impossible to complete the delivery within the time established by the teacher, but really it was not like that… each of us lived this experience to the maximum extent, trying to give everyone our best.” And another one: “It was very interesting to be able to compare face-to-face with colleagues despite the inevitable difficulties related to distance learning; however, I believe that the possibility of being able to meet teammates in-person is still the optimal solution, to better confront each other, but also to be able to view the drawings more quickly.”

This experience has allowed us an investigation on the engineering drawing aspects that can be related to the de Saussure approach, that he invented in linguistic research.

As conclusion, it can be said that Brainwriting can be conducted also not in-person and that the ideas that emerged are comparable with those when the session is performed in the classroom.
